# Gallus Heat shock cognate protein 70, a novel binding partner of Apoptin

**DOI:** 10.1186/1743-422X-8-324

**Published:** 2011-06-27

**Authors:** Kun Chen, Zheng Luo, Shijun J Zheng

**Affiliations:** 1 State Key Laboratory of Agrobiotechnology, Beijing 100193, China; 2 Key Laboratory of Animal Epidemiology and Zoonosis, Ministry of Agriculture, Beijing 100193, China; 3 College of Veterinary Medicine, China Agricultural University, Beijing 100193, China

## Abstract

**Background:**

Chicken anemia virus (CAV) infection of newly hatched chickens induces generalized lymphoid atrophy and causes immunosuppressive. VP3, also known as Apoptin, is non-structural protein of CAV. Apoptin specifically induces apoptosis in transformed or tumor cells but not in normal cells. In particular, there are no known cellular homologues of Apoptin hindering genetic approaches to elucidate its cellular function. Although a number of Apoptin-interacting molecules have been identified, the molecular mechanism underlying Apoptin's action is still poorly understood. To learn more about the molecular mechanism of Apoptin's action, we searched for Apoptin associated proteins.

**Results:**

Using yeast two-hybrid and colony-life filter approaches we got five positive yeast clones. Through sequencing and BLASTed against NCBI, one of the clones was confirmed containing Gallus heat shock cognate protein 70 (Hsc70). Hsc70 gene was clone into pRK5-Flag plasmid, coimmunoprecipitation assay show both exogenous Hsc70 and endogenous Hsc70 can interact with Apoptin. Truncated Apoptin expression plasmids were made and coimmunoprecipitation were performed, the results show the binding domain of Apoptin with Hsc70 is located between amino acids 30-60. Truncated expression plasmids of Hsc70 were also constructed and coimmunoprecipitation were performed, the results show the peptide-binding and variable domains of Hsc70 are responsible for the binding to Apoptin. Confocal assays were performed and results show that under physiological condition Hsc70 is predominantly distributed in cytoplasm, whereas Hsc70 is translocated into the nuclei and colocalized with Apoptin in the presence of Apoptin in DF-1 cell. Functional studies show that Apoptin markedly down-regulate the mRNA level of RelA/p65 in DF-1 cell. To explore the effect of Hsc70 on Apoptin-mediated RelA/p65 gene expression, we have searched two Hsc70 RNAi sequences, and found that all of them dramatically inhibited the expression of endogenous Hsc70, with the #2 Hsc70 RNAi sequence being the most effective. Knockdown of Hsc70 show Apoptin-inhibited RelA/p65 expression was abolished. Our data demonstrate that Hsc70 is responsible for the down-regulation of Apoptin induced RelA/p65 gene expression.

**Conclusion:**

We identified Gallus Hsc70 as an Apoptin binding protein and showed the translocation of Hsc70 into the nuclei of DF-1 cells treated with Apoptin. Hsc70 regulates RelA/p65 gene expression induced by Apoptin.

## Background

Chicken anemia virus (CAV) infection of newly hatched chickens causes considerable health problems and economic losses in the poultry industry worldwide. In newborn chickens CAV infection causes immunosuppression, and induces generalized lymphoid atrophy, severe anemia and increases mortality. CAV causes anemia by apoptosis of hemocytoblasts in bone marrow. Chicken thymocytes and lymphoblastoid cells can also be infected by CAV and undergo apoptosis [1].

CAV, a member of the Circoviridae family, is a small non-enveloped virus containing a single-stranded circular DNA genome of 2.3 kb. Its genome has three partially or completely overlapping open reading frames, which encode three separate proteins VP1 (51.6 kDa), VP2 (24.0 kDa) and VP3 (13.6 kDa) [2,3]. VP1 is the major viral capsid protein. VP2 is a non-structural protein with phosphatase activity of dual specificities and has been shown to interact with VP1 [4]. Both VP1 and VP2 are indispensable for CAV replication [5,6]. VP3 also named Apoptin is a non-structural protein made up of 121 amino acids, which alone can mimic the CAV-induced apoptosis in transformed chicken cells [7]. Due to the apoptotic activity of Apoptin, it has been proposed to be responsible for the apoptotic activity of whole CAV.

Apoptosis is a physiological process in which cells die in response to specific stimuli in an active, programmed manner [8]. Apoptin not only induces apoptosis in transformed chicken cells, but also can induce apoptosis of a broad range of human transformed cells or tumor cells specifically while leaving normal cells unharmed [9,10]. Apoptin induced apoptosis is p53-independent and cannot be inhibited by the anti-apoptotic proteins Bcl-2 and CrmA [11,12]. These properties of Apoptin made it an exceptional candidate for cancer therapy. The subcellular localization of Apoptin appears to be crucial for the tumor-selective apoptosis. In tumor cells, Apoptin is present predominantly in the nucleus, whereas in normal cells it remains primarily in the cytoplasm [7,13]. It is well documented that the tumor-specific nuclear accumulation of Apoptin is mediated predominantly by its C-terminal fragment, including a bipartite nuclear localization signal (NLS; amino acids 82-88 and 111-121) and a putative nuclear export signal (NES; amino acids 97-105) [9,10,14]. These recognition sequences drive the shuttling of Apoptin in and out of the nucleus. The phosphorylation of threonine^108 ^of Apoptin by the tumor-specific kinase CyclinA-CDK2 may also contribute to the regulation of its subcellular localization [15]. Apoptin also harbors a short hydrophobic leucine-rich stretch (amino acids 33-46) that is required for self-association as well as binding of promyelocytic leukemia protein (PML) and other interaction partners [2,9,16]. Recent data has linked Apoptin's complex mode of action to various cellular proteins which play prominent roles in both cell survival and death [17].

Currently, the three dimensional structure of Apoptin or its multimeric complex is not available and no recognizable cellular homologues have been found in any other species. These factors have prevented physical or genetic approaches to elucidate its function. Therefore, a yeast two-hybrid assay was adopted to search for binding partners of Apoptin which may provide important information about the molecular mechanism of Apoptin's action.

## Results

### Yeast two-hybrid Screen for Apoptin associated cellular proteins

**Figure 1:**
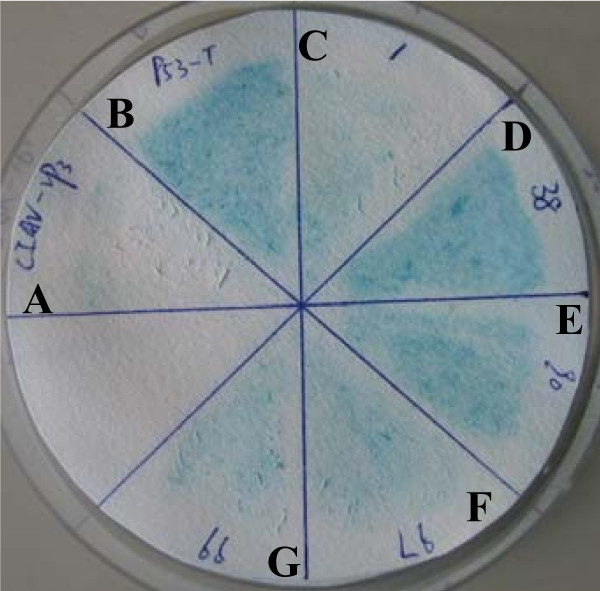
**Yeast two-hybrid screening of Apoptin associated proteins**. (A) Negative control: Yeast transformed with the β-galactosidase negative control plasmids (pGBKT7-Lam and pGADT7-T) did not turn blue. (B) Positive control: Yeast transformed with the β-galactosidase positive plasmids (pGBKT7-53 and pGADT7-T) turned blue within 20-30 min. (C)-(G) Experimental group: Yeast colonies co-transformed with the pGADT7-derivative plasmids and pGBKT7-Apoptin plasmids were checked periodically for color change.

To determine the mechanism of Apoptin's action, we screened for Apoptin interacting proteins using yeast two-hybrid approach. Negative control did not turn blue (Figure 1A) and positive control turned blue (Figure 1B). The yeast colonies, which specifically interacted with BD-Apoptin, but not BD-Null baits under both medium- and high-stringency situation, turned blue in a colony-life filter assays were considered to be positive. Approximately 1 × 10^6 ^colonies were screened for growth on SD/-Ade/-His/-Leu/-Trp medium, and the positive clones were tested for β-galactosidase activity in two additional rounds of selection. A total of five yeast clones (Figure 1C-G) yielded positive interactions. Plasmids from these clones were rescued, sequenced, and BLASTed against NCBI. Interestingly, we identified that one Apoptin-interacting protein was Gallus Heat shock cognate protein 70 (Hsc70).

### Apoptin interacts with Gallus Hsc70

**Figure 2:**
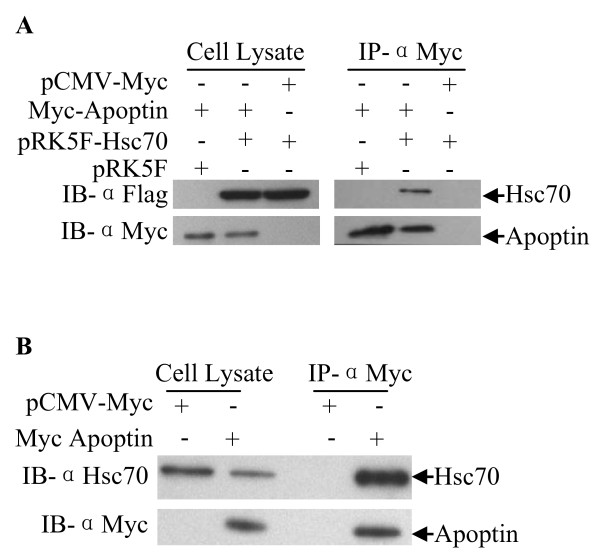
**Interaction of Apoptin with Gallus Hsc70 in DF-1 cells**. (A) Interaction of Apoptin with exogenous Hsc70 in DF-1 cells. DF-1 cells were co-transfected with plasmids encoding Myc-tagged Apoptin, Flag-tagged Hsc70, or empty plasmid (pCMV-Myc or pRK5F vector as a control) in the indicated combinations. Twenty-four hours after transfection, cell lysates were immunoprecipitated with anti-Myc monoclonal antibody and were analyzed by Western blot using anti-Myc or anti-Flag antibodies. (B) Interaction of Apoptin with endogenous Hsc70 in DF-1 cells. DF-1 cells were transfected with plasmid encoding Myc-tagged Apoptin or empty plasmid (pCMV-Myc vector as a control). Twenty-four hours after transfection, cell lysates were immunoprecipitated with anti-Myc monoclonal antibody and were analyzed by Western blot using anti-Myc or anti-Hsc70 antibodies.

**Figure 3:**
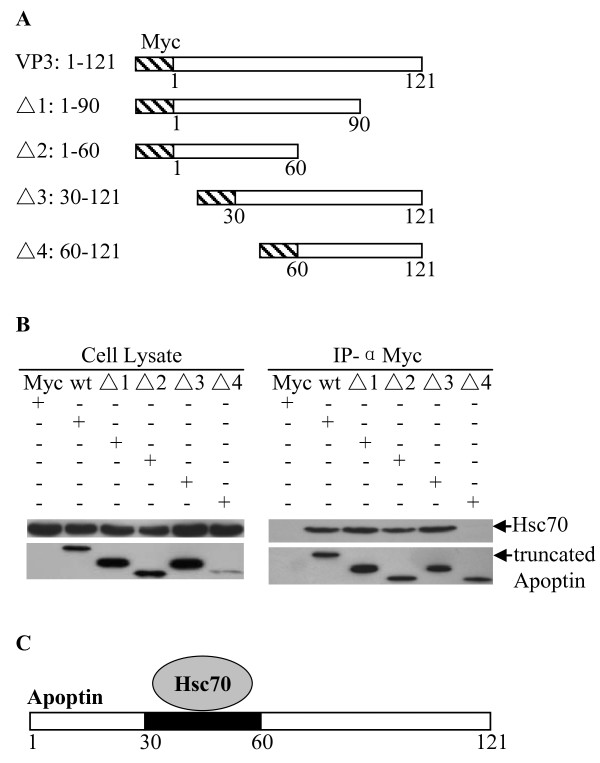
**Determination of Apoptin-binding domain for Gallus Hsc70**. (A) Schematics represent the genes encoding full length and truncated Apoptin molecules (Δ1 through 4). The numbers indicate amino acid positions within the molecule. (B) Endogenous Hsc70 interacts with different truncated Apoptin molecules. DF-1 cells were transfected with full length Myc-Apoptin (WT) or various truncated Myc-Apoptin constructs (Δ1: 1-90aa; Δ2:1-60aa; Δ3: 30-121aa; Δ4: 60-121aa). Twenty-four hours after transfection, cell lysates were immunoprecipitated with anti-Myc monoclonal antibody and were analyzed by Western blot using anti-Myc or anti-Hsc70 antibodies. (C) Schematic representation of the Apoptin's binding domain (amino acids 30-60) for Hsc70.

To explore the interaction of Apoptin with Hsc70 in a chicken cell line (DF-1) we performed an immunoprecipitation assay. pCMV-Myc-Apoptin constructs and pRK5F-Hsc70 constructs were made and co-transfected into DF-1 cells. As shown in Figure 2A, exogenous Hsc70 was precipitated from the lysate of pCMV-Myc-Apoptin transfected cells but not from pCMV-Myc-control. To examine the interaction of Apoptin with endogenous Hsc70 in DF-1 cells, pCMV-Myc-Apoptin plasmids were transfected into cells. As shown in Figure 2B, endogenous Hsc70 was precipitated by Apoptin, thus confirming Gallus Hsc70/apoptin interaction. Apoptin is a multifunction molecule comprised of several protein domains; to determine the domain responsible for Hsc70 binding, gene segments encoding fragments of Apoptin were constructed and inserted into pCMV-Myc plasmid (Figure 3A). The plasmids were transfected into DF-1 cells and immunoprecipitation assays were performed with anti-Myc monoclonal antibody. We found that all Apoptin truncations containing amino acids 30-60 interacted with Hsc70 while those without amino acids 30-60 did not (Figure 3B). These results demonstrate that the binding domain of Apoptin with Hsc70 is located between amino acids 30-60 of Apoptin (Figure 3C).

**Figure 4:**
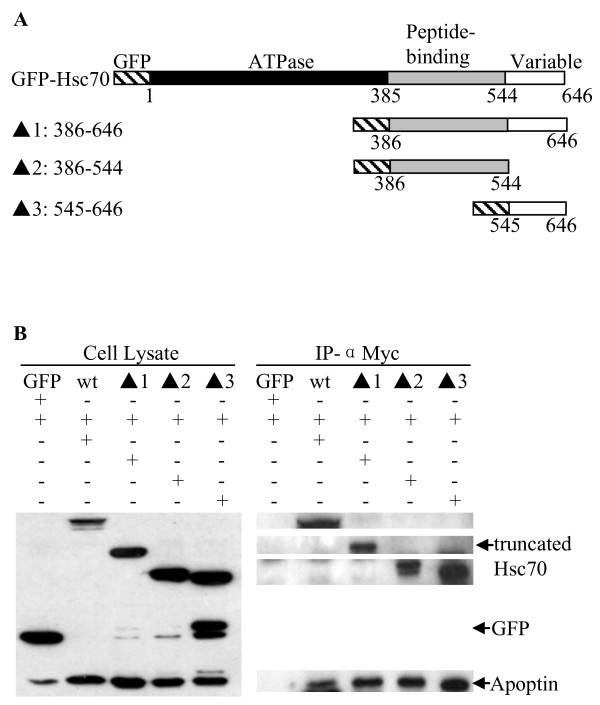
**Determination of Gallus Hsc70-binding domain for Apoptin**. (A) Schematic representation of the genes encoding full length and truncated Apoptin molecules (Δ1 through 3). The numbers indicate amino acids positions within the proteins and known functional regions are indicated. (B) Apoptin interacts with various truncated Hsc70 molecules. DF-1 cells were co-transfected with pCMV-Myc-Apoptin, full length pEGFP-Hsc70, truncated pEGFP-Hsc70 (Δ1: 386-646aa; Δ2:386-544aa; Δ3: 545-646aa) or empty pEGFP vector as a control. Twenty-four hours after transfection, cell lysates were immunoprecipitated with anti-Myc monoclonal antibody and were analyzed by Western blot using anti-Myc or anti-GFP antibodies.

Gallus Hsc70 includes three functional domains: ATPase domain (amino acids 1-385), peptide binding domain (amino acids 386-544) and variable domain (amino acids 545-646) [18]. To determine the apoptin binding domain within Hsc70, truncated Hsc70 expression plasmids (Figure 4A) were constructed and immunoprecipitation were performed. Apoptin interacts with the peptide-binding domain and variable domain of Hsc70 in DF-1 cells but not the ATPase domain (Figure 4B).

### Localization of Apoptin and Hsc70 in DF-1 cells

**Figure 5:**
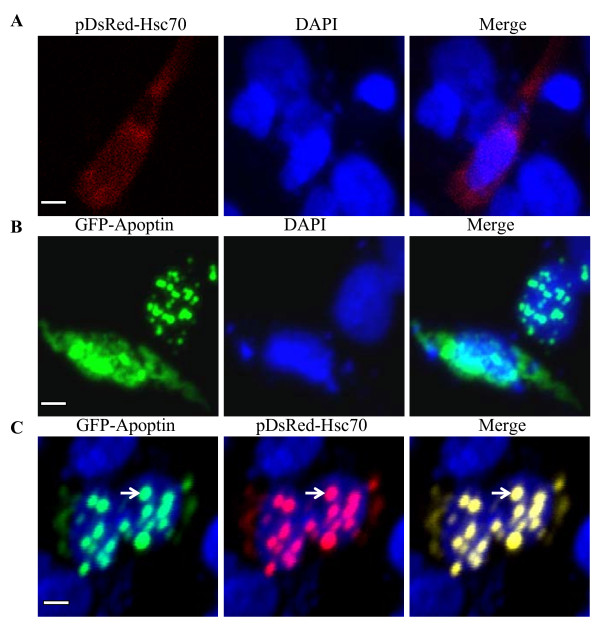
**Localization of Apoptin and exogenous Hsc70**. (A) Localization of exogenous Hsc70 in DF-1 cells. DF-1 cells were seeded on 24-well plates with coverslips and cultured overnight. The cells were transfected with pDsRed-Hsc70 plasmids. After 24 hours transfection, the cells were fixed with 1% paraformaldehyde. After washing, the fixed cells were permeabilized with 0.1% Triton X-100. Nuclei were counterstained with DAPI (Blue). (B) Colocalization of exogenous Hsc70 with Apoptin in the nucleus. DF-1 cells were transfected with a pEGFP-Apoptin plasmid and pDsRed-Hsc70 for 24 hours and treated as described above. The cell samples were observed with a laser confocal scanning microscope. The scale bars in white represent 10 μm.

**Figure 6:**
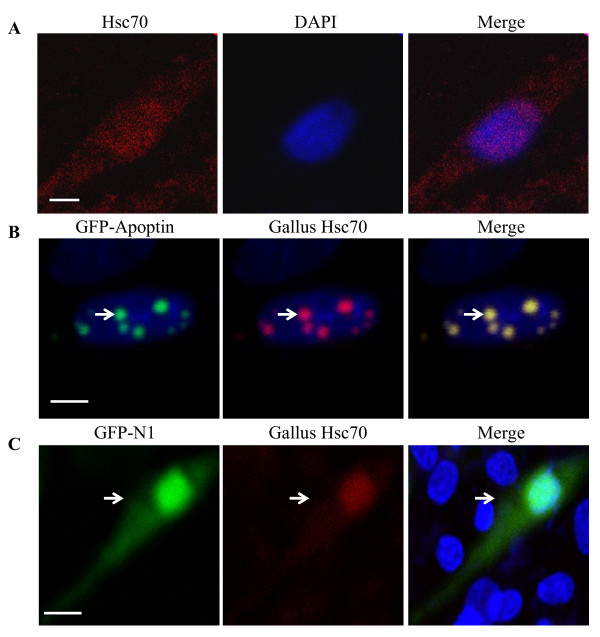
**Localization of Apoptin and endogenous Hsc70**. (A) Localization of endogenous Hsc70 in DF-1 cells. DF-1 cells were seeded on 24-well plates with coverslips and cultured overnight. The cells were fixed with 1% paraformaldehyde. After washing, the fixed cells were permeabilized with 0.1% Triton X-100, and probed with anti-Hsc70 and TRIC-conjugated secondary antibodies. Nuclei were counterstained with DAPI (Blue). (B) Colocalization of endogenous Hsc70 with Apoptin in the nucleus. DF-1 cells were transfected with a pEGFP-Apoptin plasmid for 24 hours and immunostained as described above. (C) Negative control pEGFP-N1 vector was transfected into DF-1 cells for 24 hours and immunostained as described above. The cell samples were observed with a laser confocal scanning microscope. The scale bars represent 10 μm.

To explore the localization of Apoptin and Hsc70 in DF-1 cells, we constructed pEGFP-Apoptin and pDsRed-Hsc70 expression plasmids. These plasmids were either co-transfected into DF-1 cells or transfected alone. Exogenous Hsc70 localizes to the cytoplasm (Figure 5A) and Apoptin was almost entirely distributed in or around the nucleus, which formed globular multimers (Figure 5B). When Hsc70 is co-transfected with apoptin the protein is recruited to the nucleus along with apoptin (Figure 5C). To further examine the localization of endogenous Hsc70 in the presence of apoptin, we performed immunofluorescence with DF-1 cells that were transfected with pEGFP-Apoptin expression plasmids. The distribution of endogenous Hsc70 was probed with an anti-Hsc70 monoclonal antibody, followed by a TRIC-conjugated secondary antibody. As shown in Figure 6A, endogenous Hsc70 predominantly localized to the cytoplasm of DF-1 cells. However, in pEGFP-Apoptin transfected cells, Hsc70 was translocated to the nucleus and colocalized with Apoptin in the form of granular structures (Figure 6B). However, empty pEGFP-N1 vector does not co-localize with Hsc70 (Figure 6C). These results strongly support the finding that Apoptin interacts with Hsc70 in DF-1 cells.

### Hsc70 plays an important role in Apoptin-mediated expression of NF-κB p65

**Figure 7:**
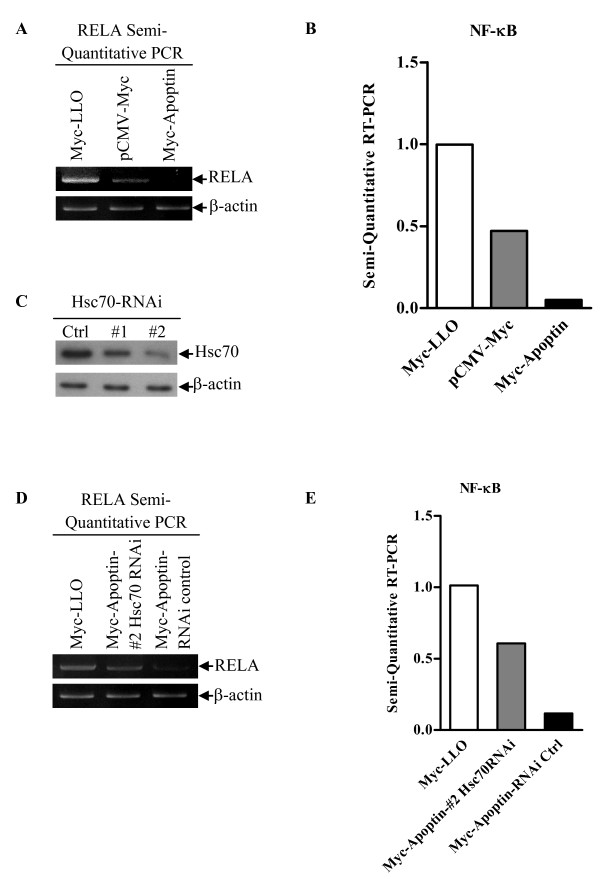
**Hsc70 inhibits Apoptin-mediated NF-κB p65 gene down-regulation**. The mRNA level of chicken NF-κB p65 in DF-1 cells was determined by semi-quantitative RT-PCR. Chicken β-actin mRNA was normalized to the RT-PCR. (A) Apoptin inhibits NF-κB p65 gene expression. DF-1 cells were transfected with pCMV-Myc-Apoptin plasmids or pCMV-Myc controls. Twenty-four hours after transfection, RNA were prepared and subjected to Semi-quantitative RT-PCR analysis. (B) The relative levels of NF-κB p65 mRNA. The density of bands in (A) was quantitated by densitometry. The relative level of NF-κB p65 mRNA was calculated as follows: the band density of NF-κB p65 mRNA/the band density of β-actin mRNA. (C) Effects of Hsc70 RNAi on the expression of endogenous Hsc70. DF-1 cells were transfected with Hsc70 RNAi (#1 and #2) or controls. Seventy-two hours after transfection, cell lysates were analyzed by Western blot with anti-Hsc70 monoclonal antibody. β-actin was used as an internal control. (D) Knockdown of Hsc70 inhibited the Apoptin caused down-regulation of NF-κB p65. DF-1 cells were transfected with the #2 Hsc70 RNAi or controls. Hsc70 RNAi treated cells were transfected with pCMV-Myc-Apoptin plasmids. Twenty-four hours after transfection, cells were lysed and subjected to semi-quantitative RT-PCR analysis. (E) The relative levels of Apoptin-mediated gene expression of NF-κB p65 in Hsc70 RNAi treated cells vs RNAi controls. The relative level of NF-κB p65 mRNA was determined as in (B).

NF-κB is a heterodimer composed of the p50 and the RelA/p65 subunits. Together the heterodimer acts as a nuclear transcription factor that plays an important role in carcinogenesis as well as the regulation of immune response and inflammatory response [19-21]. Many cancer cells show aberrant NF-κB activation, which counteracts p53-induced apoptosis by destabilizing p53 via enhanced Mdm2 expression [22]. In addition, Apoptin induces apoptosis in a p53-independent manner [11]. These data led us to ask whether NF-κB is involved in Apoptin-induced immunosuppression and apoptosis. To this question, we examined the affect of Apoptin on NF-κB p65 expression in DF-1 cells by semi-quantitative RT-PCR assay. Apoptin significantly down-regulated the mRNA level of NF-κB p65 compared to that of controls (Figure 7A and 7B). These data suggest that Apoptin inhibits gene expression of NF-κB p65 in DF-1 cells. Previous work has shown that Listeriolysin O (LLO) can strongly activate NF-κB via the IκB kinase complex, thus LLO was used as positive control [23]. To explore the effects of Hsc70 on Apoptin-inhibited expression of NF-κB p65 we first screened for Hsc70 RNAi sequences that knockdown Hsc70 and found that one fragment, #2, sequence can dramatically inhibited the expression of Hsc70 (Figure 7C). The #2 Hsc70 RNAi was used for subsequent knock-down experiments. Interestingly, we found that knockdown of Hsc70 markedly up-regulated the expression of NF-κB p65 in the presence of Apoptin (Figure 7D and 7E). These results suggest that Hsc70 plays an important role in regulating the gene expression of NF-κB p65.

## Discussion

The mechanism of CIAV-VP3 induced apoptosis and immunosuppression is not well understood. Apoptin, the vp3 gene product of CIAV is a selectively toxic to tumor cells, and is known to interact with a number of molecules within cell [17]. In this study, we used a yeast two-hybrid screen to identify Apoptin-binding partners. This screening identified several apoptin interacting proteins (data not shown), notably Gallus Hsc70 is one of them. This interaction was then confirmed by co-immunoprecipitation.

Hsc70 is a constitutive expressed chaperone that localizes primarily to the cytoplasm [18]. Transfection of apoptin in DF 1 cells, apoptin localize to the nucleus or nuclear region. Upon expression of apoptin in a DF 1 cell line, Hsc70 is translocated to the nucleus in the form of granular structures. Apoptin contains a bipartite nuclear localization sequence (NLS) and a putative nuclear export sequence (NES), which are responsible for the nucleocytoplasmic shuttling of Apoptin [9]. The amino acids 33-46 portion of Apoptin is a leucine-rich sequence that is important for its interaction with other molecules [9,10]. Hsc70 does not contain any obvious nuclear localization sequence or nuclear export sequence, so we propose that the translocation of Hsc70 from the cytoplasm to the nucleus is due to Apoptin. Our experiment finds that Apoptin interacts with Gallus Hsc70 via its 30-60 amino acids sequence. This finding supports the hypotheses of Hsc70 translocation because of Apoptin.

Hsc70 is composed of an ATPase domain, peptide-binding domain and variable domain [18]. Interaction of Hsc70 domains with Apoptin may yield different outcomes, so we investigated the binding domain of Hsc70 with Apoptin. We found that both the peptide-binding and the variable domains of Hsc70 strongly interact with Apoptin in an immunoprecipitation assay. Previous data indicated that the peptide binding domain and variable domain of Hsc70 are responsible for the stability of Hsc70-substrate complex, transport property, or interacting with other cellular or nuclear cofactors [17,18,24]. Our results suggest that the interaction of the C-terminal half (amino acids 385-646) of Hsc70 with Apoptin may assist in Hsc70's translocation into the nucleus.

Rel/NF-kB transcription factors are induced in response to many signals that lead to cell growth, differentiation, inflammatory responses, the regulation of apoptosis, and neoplastic transformation [25]. Previous data have indicated that NF-κB p65 induced AR42J pancreatoma cells proliferate despite a decrease of Hsc70 levels [26]. In addition, NF-κB plays an important role in the innate and adaptive immunity [27,28]. In newborn chickens CAV infection causes generalized lymphoid atrophy and immunosuppression, Apoptin alone can mimic CIAV's action [1]. Interestingly, we found that Apoptin can decrease the expression of NF-κB p65 in DF-1 cells; knockdown of Hsc70 abolished the inhibition of NF-κB p65 gene expression. These data suggest that the interaction of Hsc70 with Apoptin may inhibit the anti-apoptotic function of NF-κB p65 and cause immunosuppression of the host cell prior to cell death. These results may help us to understand the action of Apoptin and the CIAV induced immunosuppression in Chicken.

## Conclusions

We identified Gallus Hsc70 as an Apoptin binding partner and indicated that Apoptin interacts with Hsc70 via its 30-60 amino acids binding domain. The peptide-binding and variable domains of Hsc70 bind to Apoptin. Our results showed the nuclear translocation of Hsc70 into the nuclei of DF-1 cells treated with Apoptin. Interestingly, Hsc70 regulates the gene expression of RelA/p65 mediated by Apoptin. These findings may contribute to further understandings of the molecular mechanisms underlying Apoptin's action.

## Materials and methods

### Bacteria, cells and cDNA library

*E. coli *DH5α and DH10B were purchased from TransGen Biotech (Beijing, China) and were grown in Luria-Bertani medium. *S. cerevisiae *strain AH109 and Y187 were purchased from Clontech (USA) and used for yeast two-hybrid screen. Chicken DF-1 cells were purchased from ATCC and used for immunoprecipitation and confocal assay. Chicken spleen cDNA expression library was constructed by Takara Biotechnology (Dalian, China) and used to screen Apoptin binding partners.

### Reagents

The restriction enzymes (*Bgl *II, *Xho *I, *Bam*H I, *Sal *I, *Eco*R I, *Xba *I, *Hind *III, and *Not *I), reagent for reverse transcription Oligod(T)_15, _RNase inhibitor, 10 mM dNTPs were purchased from Takara Biotechnology (Dalian, China). pCMV-Myc vector, pRK5F vector, pDsRed-monomer-N1 vector and pEGFP-N1 vector were purchased from Clontech (USA). The Large Amount Without Endo-toxin Plasmid Preparation Kits was purchased from Aidlab (Beijing, China) and was used to prepare plasmids per manufacturer's instruction. Fetal bovine serum of Hyclone was purchased from BeiFangTongZheng (Beijing, China). Dulbecco's modified eagle's medium high glucose was purchased from Invitrogen (USA). SD/-Trp, SD/-Leu, SD/-Ade/-His/-Leu/-Trp medium and β-galactosidase (β-Gal or X-Gal) were purchased from Clontech (USA). PVDF Membrane was purchased from Millipore (USA). Mouse monoclonal IgG1 antibody against c-Myc (9E10) SC-40, mouse monoclonal IgG2a antibody against GFP (B-2) SC-9996, and protein A/G plus-agarose SC-2003 for immunoprecipitation were purchased from Santa Cruz Biotechnology (USA). Mouse monoclonal anti-Flag M2 antibody IgG1 (F1804) was purchased from Sigma (USA), Anti-rat monoclonal antibody IgG2a of Hsc70 (1B5, ab19136) was purchased from Abcam (Europe). TRITC (Rhodamine-Conjugated Affini Pure Goat Anti-Rat IgG), horseradish conjugated goat-anti mouse polyclonal antibodies and horseradish conjugated goat-anti rat polyclonal antibodies were purchased from DingGuoShengWu (Beijing, China). 4', 6-diamino-2-phenylindole (DAPI) was purchased from Beytime (Nanjing, China).

### Plasmids construction

The pcDNA4.0-vp3 plasmids constructed by Huang ChaoHua of our lab were used as template to amplify the vp3 gene. To generate the pCMV-Myc-Apoptin plasmid the sense primer 5'-CGAATTCATGAACGCTCTCCAAG-3' and the anti-sense primer 5'-GCGGCCGCTTACAGTCTTATACGCCTTCT-3' were used, the gene segments were digested with *Eco*R I and *Not *I and cloned into pCMV-Myc in frame. When constructing the pGBKT7-Apoptin plasmid the vp3 gene was cloned using the sense primer 5'-GAATCCATGAACGCTCTCCAAGA-3' and the anti-sense primer 5'-GGATTCTTACAGTCTTATACGCCTTCT-3' and cut with *Eco*RI and *Bam*HI and ligated downstream of the gene encoding the yeast GAL4 DNA-binding domain (BD). The plasmid pEGFP-Apoptin was constructed with the sense primer 5'-GCGAATTCTGATGAACGCTCTCCAAGAAGAT-3' and anti-sense primer 5'-GCGGATCCCGCAGTCTTATACGCCTT-3'. The Apoptin deletion mutants of, △1 pCMV-Myc-vp3 (1-90aa), △2 pCMV-Myc-vp3 (1-60aa), △3 pCMV-Myc-vp3 (30-121aa) and △4 pCMV-Myc-vp3 (60-121aa) were constructed with the following primers: for △1 the sense primer is 5'-GCTTGAATTCGGATGAACGCTCTCCAAG-3' and the anti-sense primer is, 5'-GCTTCTCGAGATTAGCAGGATCGC-3', for △2 the sense primer is 5'-GCTTGAATTCGGATGAACGCTCTCCAAG-3' and the anti-sense primer is 5'-GCTTCTCGAGATTATGCAGATCTTAG-3', for △3 the sense primer is 5'-GCTTGAATTCGGCCTCACTGCAGAGA-3' and the anti-sense primer is 5'-GCTTCTCGAGATTACAGTCTTATACG-3', for △4 the sense primer is 5'-GCTTGAATTCGGACTGCGGACAATTC-3' and the anti-sense primer is 5'-GCTTCTCGAGATTACAGTCTTATACG-3'. The PCR products were digested with *EcoR*I and *Xho*I respectively. The Gallus gene segments coding for full length Hsc70 (Gene ID: 395853) were cloned by standard molecular cloning procedures and cloned into the pRK5F vector to generate pRK5F-Hsc70 plasmids encoding Flag-tagged Hsc70. The primers for full length of Hsc70 of chicken DF-1 cell are sense 5'-TCTAGAATGTCAAAGGGACCAGCTGT-3' and anti-sense 5'-AAGCTTTAAATCCACCTCCTCAATG-3'. Gallus pEGFP-hsc70 and pDsRed-Hsc70 plasmids were separately constructed by PCR using Gallus pRK5F-Hsc70 as template and with sense primer 5'-GCAGATCTCATGTCAAAGGGACCAGCTGT-3' and anti-sense primer 5'-GCGTCGACTGATCCACCTCCTCAATGGTT-3'. The PCR products were cut with *Bgl*II and *Sal*I. The Gallus Hsc70 deletion mutants of, ▲1 pEGFP-Hsc70 (386-646aa), ▲2 pEGFP-Hsc70 (386-544aa) and ▲3 pEGFP-Hsc70 (545-646aa) were generated by PCR using Gallus pRK5F-Hsc70 as template. For ▲1 pEGFP-Hsc70 the sense primer is 5'-GCTTCTCGAGATGGAGAATGTTCAAGATTTGCT-3' and the anti-sense is 5'-GCTTGGATCCCGATCAACCTCTTCAATGGTG-3'; for ▲2 pEGFP-Hsc70 the sense is 5'-GCTTCTCGAGATGTATGCCTTCAACATGAAAG-3' and the anti-sense is 5'-GCTTGGATCCCGATCAACCTCTTCAATGGTG-3'; for ▲3 pEGFP-Hsc70 the sense is 5'-GCTTGGATCCATGGAGAATGTTCAAGATT-3' and antisense is 5'-GCTTAAGCTTTTAGGACTCAAGTGAATTC-3'. The PCR products were cut with *Xho*I and *Bam*HI respectively. All the primers were synthesized by Augct Company (Beijing, China) and all the plasmids were confirmed by sequencing.

### Yeast Two-Hybrid Screen and Colony-life Filter Assay

Yeast two-hybrid screen was performed according to the manufacturer's protocol (Matchmaker Two-Hybrid System 3). The pGBKT7-Apoptin plasmid expressing the fusion protein GAL4-BD-Apoptin was used as bait and transformed into *Saccharomyces cerevisiae *AH109 which is Trp¯ and contains *HIS3, ADE2 *and *LacZ *reporter genes for GAL4 transcriptional activity. Chicken cDNA expression library fusion to the GAL4-activation domain in the pGADT7 plasmid was transformed into the *Saccharomyces cerevisiae *strain Y187 which is Leu¯ and contains the *lacZ *reporter genes. In β-Gal colony-life filter assay, the bait plasmid was shown to have no transactivation activity. Next, the cDNA Library clones expressing the interacting prey proteins were screened by yeast mating. Positive clones were selected on SD/-Ade/-His/-Leu/-Trp medium and tested for β-galactosidase activity (LacZ^+^) by colony-lift filter assay. The p53 and large T-antigen were used as positive controls. The pGBKT7-53 and pGADT7-T encode a fusion of the GAL4 DNA-BD and AD and murine p53 and SV40 large T-antigen, respectively. Yeast transformed with the β-galactosidase positive plasmids turned blue within 20-30 min. Lam and the large T-antigen was used as a negative control. The pGBKT7-Lam and pGADT7-T encode a fusion of the GAL4 DNA-BD and AD and Lam and SV40 large T-antigen, respectively. Yeast transformed with the β-galactosidase negative control plasmids did not turn blue. Yeast colonies co-transformed with the pGADT7-derivative plasmids and pGBKT7-Apoptin plasmids were checked periodically for the appearance of blue colonies, typically from 30 min to 8 h. The double-positive colonies (His^+^/LacZ^+^) were selected for rescue of plasmids in *E. coli *strain DH10B.

The resulting clones were sequenced with the GAL4-AD sequencing sense primer 5'-AGATGGTGCACGATGCACAG-3' and the results were BLASTed against the NCBI database.

### RNA extraction, cDNA synthesis, and RT-PCR

Total RNA was extracted from 14 day chicken spleen tissue by standard molecular clone techniques using TRIZOL reagent (Invitrogen, USA) per the manufacturer's instructions. One microgram of total RNA was incubated with 0.5 μg of Oligod(T)_15 _for 5 min at 70°C to melt the secondary structure before cooling immediately on ice. RNA was subsequently combined with 40 U of RNase inhibitor, 10 mM dNTPs and 200 U of Moloney murine leukemia virus reverse transcriptase (Promega, USA) in reverse transcriptase buffer in a total volume of 25 μL before incubation at 37°C for 60 min. To enable appropriate amplification in the exponential phase of the target gene, PCR amplification of Gallus genes were conducted with specific primers.

### Cell Culture and transfection

Cells were cultured in DMEM high glucose with 10% fetal bovine serum and non-essential amino acid solution and were maintained in 5% CO_2 _at 37°C. 8 × 10^5 ^cells were seeded into 6-well plate and plasmids were transiently transfected into the cell after 24 h with the Vigofect transfection reagent (Vigorous Beijing, China) according to the manufacturer's protocol.

### Immunoprecipitation and Western Blot assay

The interaction of Apoptin with exogenous Hsc70 was identified by co-immunoprecipitation. 8 × 10^5 ^cells DF-1 cells were plated into 6 well plates the day before transfection. 2.5 μg pRK5F-Hsc70 and pCMV-Myc-Apoptin or empty vector (pRK5F or pCMV-Myc as a control) was transfected into cells. 24 h after transfection, whole cell lysates were prepared with 0.2 mL lysis buffer (50 mM Tris-HCl, pH8.0, 150 mM NaCl, 5 mM EDTA, pH8.0, 1 mM DTT, 1% NP-40, 10% Glycerol and 1 × complete protease cocktail inhibitor) at 4°C for 30 min. For immunoprecipitation, the lysates were incubated with 2 μg of an anti-mouse c-Myc monoclonal antibody at 4°C for 2 h, then mixed with 20 μL of 50% slurry of protein A/G plus-agarose and incubated for an additional 2 h. Beads were washed three times with lysis buffer and boiled with 2 × SDS loading buffer for 10 min. Protein samples were resolved by SDS-PAGE and transferred onto PVDF membrane, the membrane was blocked with 5% skim milk. These samples were probed with anti-Flag M2 monoclonal antibody or c-Myc monoclonal antibody and detected using the ECL chemiluminescence kit.

The interaction of Apoptin with endogenous Hsc70 was confirmed with co-immunoprecipitation as described above. The samples were probed with Hsc70 monoclonal antibody and c-Myc monoclonal antibody and detected using the ECL chemiluminescence kit.

### Subcellular localization of the two proteins in cells

The day before transfection 4 × 10^5 ^DF-1 cells were plated onto 6 wells-plates with glass coverslips in it, 1 μg of pEGFP-Apoptin plasmid and 2 μg of pDsRed-Hsc70 plasmid were either co-transfected or transfected alone. 24 h after transfection cells were fixed with 4% paraformaldehyde for 15 min, washed with pH7.4 PBS three times, and the nuclei were counterstained with 4', 6-diamino-2-phenylindole (DAPI, 10 μg/mL) for 1 min before washing with PBS pH7.4 three times. To examine the endogenous Hsc70, the cells cultured on coverslips were fixed with 4% paraformaldehyde for 15 min, washed with PBS pH7.4 thee times, permeabilized with 0.2% Triton X-100/PBS for 15 min, washed with pH7.4 PBS three times and blocked in 1% BSA/PBS for 30 min. Monoclonal antibody of Hsc70 in 1% BSA/PBS was added and incubated for 1 h before washed with pH7.4 PBS three times. A secondary TRITC-conjugated goat-anti-rat polyclonal antibody was used for detection. 1 h after washing with PBS pH7.4 three times, counterstained with DAPI for 1 min, and mounted with fluorescent mounting solution (DAKO). Images were analyzed using Nikon C1 Standard Detector (Japan) equipped with UV, TRITC, and FITC/GFP filter sets. The overlay of fluorescent images was performed using EZ-C1 software.

### Truncation analysis of the binding region of Apoptin to Gallus Hsc70

Plasmids expressing different Gallus Hsc70 deletion mutants were generated as described above. Interaction of Apoptin with Gallus Hsc70 truncated proteins was assessed by immunoprecipitation assay as described above. The samples were immunoblotted with anti-GFP and anti-c-Myc monoclonal antibody then detected using ECL chemiluminescence kit.

### Truncation analysis of the binding region of Gallus Hsc70 to Apoptin

Apoptin deletion mutants were generated as described above. Interaction of Gallus endogenous Hsc70 with Apoptin truncated proteins was assessed by immunoprecipitation assay as described above. The samples were probed with anti-c-Myc and anti-Hsc70 monoclonal antibody and detected using ECL chemiluminescence kit.

### RNAi screening

All small interfering RNAs (siRNAs) against Gallus Hsc70 and Silencer negative control siRNA were purchased from Shanghai Genechem (China). Lipofectamin RNAiMAX (Invitrogen) was used to transfect siRNAs into the DF-1 cells, according to the manufacturer's recommendations. The sense sequences of the siRNAs are as follows: siRNA1, 5'-GCGCAAGCACAAGAAAGACATCAGT-3'; siRNA2, 5'-GGACAACAACTTGCTGGGCAAGTTT-3'. Seventy-two hours after transfection, cells were harvested for Western blot analysis.

### Gene expression analysis

The expression of Gallus NF-κB p65 was detected by semi-quantitative RT-PCR. 24 h post transfection, total RNA was extracted from DF-1 cells using Trizol Reagent (Invitrogen, USA) and treated with DNase-I, amplification grade (Invitrogen, USA) according to manufacturer's protocols. 1 μg of total RNA was used for cDNA synthesis using the SuperScript™ III Reverse Transcriptase kit (Invitrogen, USA) according to the manufacturer's protocol. For PCR, 50 ng of the cDNA sample was added to a 25 μl PCR mixture. PCR conditions were as follows: 94°C for 30 sec, 55°C for 30 sec, and 72°C for 10 sec. After 30 cycles, equal volumes of the resulting PCR reactions were analyzed by electrophoresis on a 1.5% agarose gel. The densitometric analysis of bands was performed using Alpha Imager software. Chicken β-actin was used as an internal control. Gallus NF-κB p65 (Gene ID: NM_205129.1) primers, sense primer 5'-ACGAGTTGGTGGGCCGCCATTG-3' and anti-sense primer 5'-CACGGTTGTCATAGATGGGCT-3'. Gallus β-actin (Gene ID: NM_205518.1) primers, sense primer 5'-ATTGAACACGGTATTGTCACCA-3' and anti-sense primer 5'-TAGCCTTCATAGATGGGCACA-3'.

## Abbreviations

CAV: Chicken anemia virus; Hsc70: Heat shock cognate protein 70.

## Competing interests

The authors declare that they have no competing interests.

## Authors' contributions

All authors read and approved the final manuscript. All authors have equal contribution to this paper.

## Author details

College of Veterinary Medicine, China Agricultural University, 2 Yuan-Ming-Yuan West Road, Beijing 100193, China
